# Effects of Peroneus Brevis versus Peroneus Longus Muscle Training on Muscle Function in Chronic Ankle Instability: A Randomized Controlled Trial

**DOI:** 10.3390/healthcare12050547

**Published:** 2024-02-26

**Authors:** Dukhan Ko, Yongchul Choi, Kyujin Lee

**Affiliations:** 1 Department of Sports Science Convergence, Dongguk University, Seoul 04620, Republic of Korea; kodh119@hanmail.net; 2 Department of Physical Education, Gangneung-Wonju National University, Gangneung 25457, Republic of Korea; 3 Department of Instructor Education, College of Cultural Convergence, Jeonju University, Jeonju 55070, Republic of Korea

**Keywords:** chronic ankle instability, peroneus brevis muscle, peroneus longus muscle, strength, Y-balance test

## Abstract

Chronic ankle instability (CAI) is a common injury that can occur in daily life or sporting events. Injuries to the anterior talofibular, posterior talofibular, and calcaneofibular ligaments are common, and the core of rehabilitation training involves strengthening the peroneus muscle. Many studies on rehabilitation training have focused on strengthening the peroneus brevis muscle, and few studies have focused on specific training to strengthen the peroneus longus muscle. Therefore, this study aims to investigate changes in the symptoms and functions of patients by applying training to strengthen the peroneus longus and peroneus brevis muscles. Home-based training and mobile monitoring were utilized for 12 weeks, divided into peroneus brevis training (PBT) and peroneus longus training (PLT), in 52 adult males with CAI. Participation was voluntary, with enrollment done through a bulletin board, and intervention training allocation was randomly assigned and conducted in a double-blind manner. This study was registered as a trial protocol (KCT 0008478). Foot and ankle outcome scores (FAOS), isokinetic ankle strength tests, and Y-balance tests were performed before and after the intervention. Both PLT and PBT significantly improved in FAOS, inversion, and eversion at angular velocities of 30°/s and 120°/s and in the anterior and posterolateral directions of the Y-balance test (*p* < 0.05). Interaction effects by time and group were not significant for the FAOS (*p* > 0.05). However, PLT improved eversion muscle strength and muscle power to a greater degree, compared with PBT, in the anterior and posterolateral directions of the Y-balance test (*p* < 0.05). In conclusion, both PLT and PBT were effective for CAI patients; in addition, PLT had greater potential for improving strength and balance.

## 1. Introduction

The ankle joint is the farthest joint from the center of the human body and bears the highest weight-bearing burden. In addition, because it is a joint that directly contacts the ground while walking, it is responsible for the mobility of the human body and requires stability during activities [1]. Another characteristic of the ankle joint is its high risk of injury; 90% of ankle injuries are inversion ankle sprains. This injury first appears in the form of acute trauma but is characterized by becoming chronic, and this is called chronic ankle instability (CAI) [2,3].

According to one study [2], 23.4% of the surveyed athletes had CAI, and retrospective and prospective studies have reported that 30–40% of patients with ankle sprains develop CAI [4]. The causes of CAI are attributed to the following anatomical features. The ankle joint descends slightly longer than the lateral malleolus, limiting the eversion of the foot. However, as the medial malleolus is relatively short, it has a large range of motion for inversion. Consequently, it is vulnerable to inversion ankle sprains when the ankle is injured [5]. The talus connects the tibia at the top and the calcaneus at the bottom. It does not provide an attachment point for the muscles and there is no regularity when viewed from any direction. Owing to the atypical structure of the talus, it has an advantage in terms of mobility but a disadvantage in terms of stability [6,7]. Finally, the ankle joint is also vulnerable to injury as its axis of motion is oblique [8]. When viewed from the frontal and transverse planes, the axis of motion of the ankle joint has a diagonal structure; it undergoes slight inversion and eversion during dorsiflexion and plantarflexion [1]. Therefore, the ankle joint is inevitably vulnerable to damage when the weight is supported on an irregular floor. This is particularly dangerous when the ankle is inverted and is in contact with the ground [9].

Another cause of chronic ankle instability, other than the anatomical substrate described above, is lack of proprioception in ligaments that have been loosened due to sprains. This causes loss of ‘position sense’ and increases the risk of re-injury [10]. Foot inversion injury damages the anterior talofibular ligament (ATFL), posterior talofibular ligament (PTFL), and calcaneofibular ligament (CFL). One study reported that ATFL damage alone comprised 50% of injuries, and the prevalence of ATFL damage accompanied by CFL was 20–30% [11]. In cases of ATFL damage alone, conservative treatment or rehabilitation is recommended over surgery [12]. For ATFL-only injuries, conservative or rehabilitation treatment, rather than surgery, is recommend, and peroneus muscle strength training is emphasized [13].

The peroneus is divided into two muscles: the peroneus longus (PL) and peroneus brevis (PB). These two muscles contribute 70% of the eversion action and compensate for the function of the ATFL, PTFL, and CFL ligaments, which can no longer function following lateral ligament rupture or sprains [14]. Because of differences in length and anatomical function, the two muscles may act differently depending on the position of the ankle. According to electromyographic studies, strength training in PL was reported to be associated with eversion in ankle plantar flexion, while PB was associated with eversion in a 90° neutral ankle position [15,16,17]. In general, rehabilitation after CAI injury as a conservative therapy involves ankle eversion strength training using bands to train these muscles [18]. However, ankle eversion muscle strengthening is primarily performed with a band tied in a neutral position, with an ankle angle of 90°and the PB acting as the main muscle, and training in the plantar flexion position is relatively rare [19]. However, the most common posture for sprains in patients is when the foot is inverted in a slightly plantar-flexed position; eversion training should be performed with the ankle in the plantarflexion position to mechanically strengthen the PL muscle.

Therefore, this study investigated the eversion training effect of a neutral ankle position and a plantarflexion position. The hypothesis presented in this study was that there would be a significant difference between eversion training in ankle plantar flexion and eversion training in the neutral position in CAI patients.

## 2. Methods

### 2.1. Participants

The sample size was calculated using G*power software (G*power 3.1.9.4, University of Düsseldorf, Düsseldorf, Germany): α error prob = 0.05; power, (1−β err prob) = 0.95; and effect size f = 0.25 (medium). To establish the effect size, we referred to another study that administered 12 weeks of training [20,21]. The calculation results in the total sample size = 54, the critical F = 4.026, and denominator df = 52.0.

The participants in this study were males diagnosed with CAI following their visit to the orthopedic department of a hospital in Seoul, Korea, and who voluntarily participated after looking at the bulletin board. Written informed consent for this study was obtained from all participants prior to study enrolment. In addition, the possibility of benefits and harms (ex, discomfort) from participating in the study was explained, and participants were notified that they could stop participating at any time they wanted. This study was approved by the Research Ethics Committee of Gangneung-Wonju National University (approval number 2021-13). The trial protocol number for this study is KCT0008478 (Supplementary Table S1).

The participants were patients who complained of ankle pain for at least 6 months. Patients who underwent surgery and those in the inflammatory phase after acute injury were excluded. Fourteen patients were excluded due to lost follow-up and discontinue of intervention training. For each intervention, 27 patients participated in PLT, 27 participated in PBT, and 2 patients dropped out, resulting in a final analysis of 52 patients. The primary outcome is strength and balance, and the secondary outcome is the FAOS evaluation using a questionnaire.

Because the design was a parallel group randomized controlled trial and handled in a double-blind manner, the investigators were not involved in intervention allocation, which was conducted by an assistant. Additionally, researchers and examiners were blinded to participants’ intervention group information in tests conducted before and after the intervention.

The allocation process is as follows. After listing participants in order of center registration number, a random number table was used. Afterwards, using the last digits of the random number table, odd numbers were designated as PBT and even numbers were designated as PLT. All of these processes were carried out by an assistant, and the researcher was not involved. Group assignments were performed by non-research staff to exclude the opinion of researchers. Participants’ general characteristics are shown in Table 1.

### 2.2. Research Procedure

The study participants were classified into PBT and PLT groups using the allocation method. The participants underwent training at home as they received training education and feedback, and Q & A sessions were provided using a mobile device. The intervention period was 12 weeks, and participants were instructed to train twice a day, in the morning and evening, and the training intensity was adjusted to a level of 11 (normal) to 13 (slightly difficult) using Rating Perceived Exertion (RPE). The participants were instructed to train within a pain-free range. In the event of any discomfort, they were educated on the importance of reducing training intensity, and mobile access was provided for assistance.

**Figure 1:**
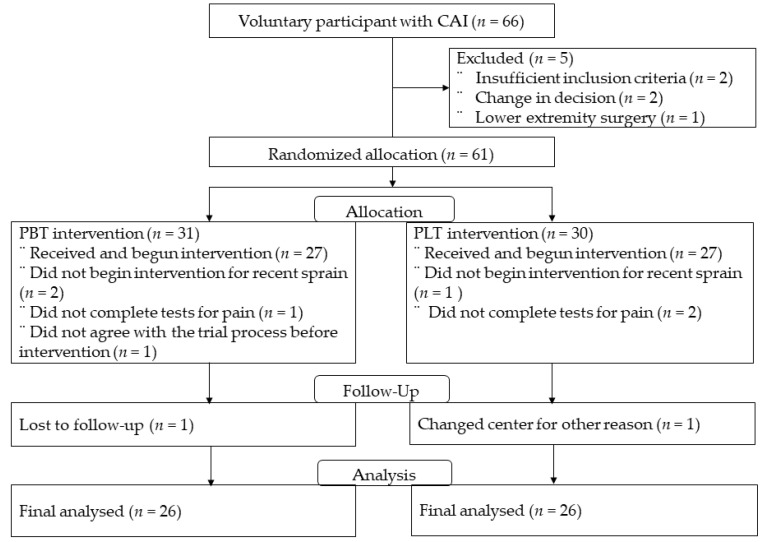
Participant’s inclusion and exclusion. CAI, chronic ankle instability; PBT, proneous brevis training; PLT, proneous logus training.

The total training time was approximately 30 min, and the patient was required to perform 20–30 min of stationary bicycle training daily to alleviate intrinsic ankle pain. The participants revisited the hospital after 12 weeks to be examined for the effect of training, which consisted of at least 60 min per day. The foot and ankle outcome score (FAOS), ankle isokinetic inversion and eversion test, and Y-balance test were used to evaluate subjective ankle pain before and after the intervention. The overall study design is shown in Figure 1.

### 2.3. Foot and Ankle Outcome Score

The foot and ankle outcome score (FAOS) was used to comprehensively evaluate the participants’ ankle pain, discomfort perceived in daily life and sports situations, and quality of life related to CAI [22]. The questionnaire consisted of five items: pain, symptoms, daily activity, sports, recreational physical activity, and quality of life. There were 42 questions, and a subjective self-assessment was performed using a scale from 0 to 4 points. The highest score for each item was converted to 100 points. The higher the score, the better the condition; the lower the score, the greater the discomfort.

### 2.4. Isokinetic Ankle Joint Function Test

Ankle isokinetic muscle strength was measured using a CSMi dynamometer and HUMAC software (CSMi HUMAC NORM, Stoughton, MA, USA, 2015). Isokinetic muscle strength measurement is widely used in clinical practice because it expresses muscle contraction according to a computer-controlled speed within the normal range of motion of a joint [23]. Ankle inversion and eversion were measured, and the test was adopted in concentric contraction mode. The test angle was set from inversion 50° to eversion 20° based on the neutral position. The temperature of the examination room was maintained at 22–26 °F, and the participants were instructed to avoid high-intensity training or excessive meals before the examination to ensure the best conditions. The angular velocity was set at 30°/s for muscle strength measurement (Nm) and 120°/s for muscle power measurement (Watt). Preparation and practice continued until the participant was familiar with the test equipment and method; the actual test was repeated four times.

Considering the stability of the test, a healthy ankle was first examined, and the injured ankle was measured subsequently. The maximum value of the injured ankle was recorded. For both muscle strength and power measurements, the measured value was divided by the body weight to compensate for the weight variable.

### 2.5. Y-Balance Test

**Figure 2:**
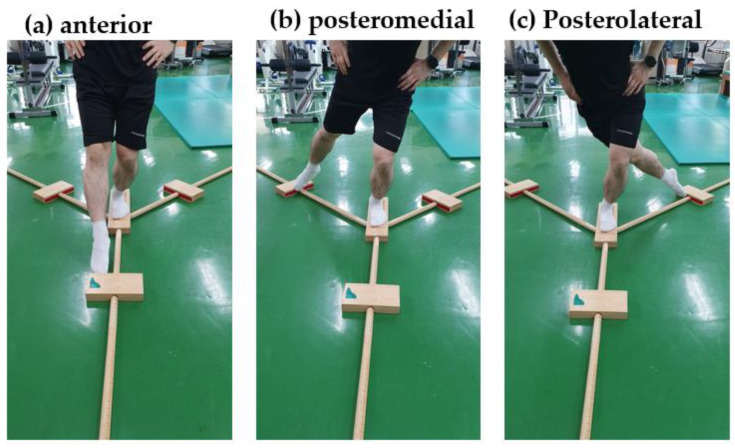
Y-balance Test.

Dynamic balance was measured using the Y-balance test (FMS Inc., Chatham, VA, USA) (Figure 2). The Y-balance test was developed from the previous 8-direction Star excursion balance test. Three directions (*anterior*, *posteromedial*, and *posterolateral*) with high reliability were selected and tested to evaluate dynamic ankle balance and proprioception [24,25]. Each participant listened to a sufficient explanation before the test, which was conducted approximately thrice. The participants placed one foot on the examination table and secured it in place. Using the unfixed foot, the patient stretched out as far as possible in the anterior, posterior medial, and posterior lateral directions and measured the distance. The measuring scaffold was adjusted to its maximum extent, and the distance was measured in units of 0.1 cm, repeated three times in each direction. The test was repeated if the measuring scaffold was pushed due to recoil or if the posture was not maintained for more than 2 s. The *Y-balance test score* was calculated using a standardized formula based on the participant’s *leg length* and three directions. The lower limb extends from the anterior superior iliac spine to the medial malleolus. The following formula was used:

(1) $$Y-balance\;test\;score=\frac{anterior+posteromedial+posterolateral}{leg\;length\times 3}\times 100$$

### 2.6. Intervention Training

**Figure 3:**
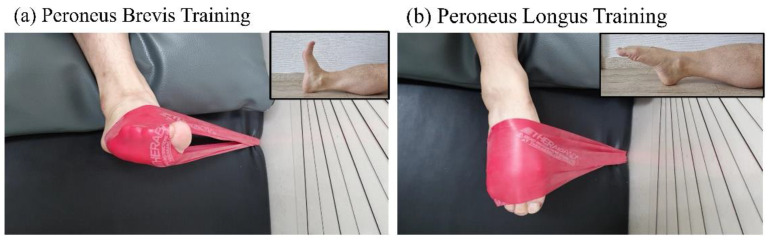
Peroneus brevis training (**a**) and peroneus longus training (**b**).

Each session of PBT and PLT intervention training lasted approximately 50 min. This consisted of 10 min of ankle stretching, 20 min of stationary cycling, and 20 min of ankle band exercise for each group (PBT, PLT). The Theraband^®^ (Hygenic Corp., Akron, OH, USA) was used for the intervention. The PBT Program refers to typical ankle eversion training. This movement changed the ankle angle from the neutral position of 90° (Figure 3a). The PLT Program is an operation that facilitates the evolution of the ankle in the plantarflexion posture (Figure 3b).

In the early stages of rehabilitation, the participants performed eversion 20 times for 5 s each without applying a band to understand and familiarize themselves with the range of joint motion, muscle activation, and posture. After 1 week, if there was no pain or swelling caused by training, resistance training was initiated by using an elastic band. Training started with 10 sets of 15 repetitions, and the training volume increased gradually. The color of the band started with red and strengthened to green and blue. After 4 weeks, the participants visited the clinic again for a mid-term checkup, received interim education, and adjusted the training intensity and training volume.

### 2.7. Data Analysis

Statistical analysis was performed using IBM SPSS Statistics, version 25.0 (IBM Corp., Armonk, NY, USA). The dependent variables were continuous variables, and a normality test was performed using the Shapiro–Wilk test. Data are expressed as mean and standard deviation, and comparisons between groups were performed using an independent *t*-test. A paired *t*-test was performed for comparison before and after the intervention within the group, and a repeated measures two-way ANOVA was applied to confirm the interaction effect according to group and time. The formula for calculating the difference between the dependent variable values before and after the intervention was as follows:

(2) $$Difference\;\left(\%\right)=\frac{post-pre}{pre}\times 100$$

Statistical significance was set at *p* < 0.05.

## 3. Results

### 3.1. Changes in Foot and Ankle Outcome Score According to Training

Table 2 presents the subjective self-assessments before and after the intervention. Pain and symptoms associated with CAI and discomfort during daily and sports activities significantly improved after training in both the PBT and PLT groups (*p* < 0.05). However, differences were not observed between the groups over time.

### 3.2. Changes in Isokinetic Ankle Joint Function According to Training

In the isokinetic muscle strength test after the intervention, both the PBT and PLT showed significant improvement after 30°/s inversion, eversion, and 120°/s eversion (*p* < 0.05). The effects of the time and group were significant at 30° and 120°/s. There were no significant differences between the groups at the 30°/s and 120°/s inversions (Table 3).

### 3.3. Changes in the Y-Balance Test According to Training

Table 4 summarizes the results of the Y-balance test for ankle stability after 12 weeks of the training program. After PBT, the anterior, posteromedial, and Y-balance scores increased significantly (*p* < 0.05). In the PLT, all items, such as anterior, posteromedial, posterolateral, and Y-balance scores, increased significantly. Regarding the effect according to time and group, the anterior and posteromedial regions differed significantly (*p* < 0.05).

## 4. Discussion

This study aimed to identify the differences between isolated rehabilitation exercises that strengthen the peroneus brevis and longus muscles in patients with CAI. CAI is traditionally attributed to tears or looseness in the ATFL and CFL [26]. To compensate for this, the core of rehabilitation is to restore the function of the damaged ATFL and CFL by strengthening the peroneus brevis muscle and peroneus longus muscles located outside the fibula. It was suggested that the reason for this is that the evertor becomes very weak in patients with CAI, and this phenomenon induces elongation and contributes to more inversion of the foot when walking [27].

In this study, the FAOS demonstrated a significant improvement after the intervention in both groups. However, when comparing the pain and symptom relief effects between the groups, PLT did not yield statistically significant results compared with PBT. Several previous studies have demonstrated the effectiveness of rehabilitation training using bands as a conservative treatment for patients with CAI. In a previous study, band, balance, and combination training were performed for four weeks by 43 elite youth athletes with CAI. Improvements were observed in the athletes’ ability measurement scores [28]. Another study reported the effect of band training in 21 patients with CAI, finding that the patients’ pain scale scores decreased as muscle strength increased [29]. This study also demonstrated a consistent trend that aligns with the results of previous studies.

Against this background, a difference in muscle activity was expected. A comparison of electromyography (EMG) findings of the two muscles according to ankle posture was previously researched [17,30]. The study found that the peroneus longus muscle activity was significantly higher in the eversion motion in plantar flexion than it was in the neutral position. The advantage of performing eversion training in the plantarflexion posture is that it also activates the peroneus brevis muscle significantly. Therefore, incorporating both plantar flexion and ankle eversion training can offer additional benefits to patients with CAI because it trains the peroneus longus and the peroneus brevis muscles.

In the isokinetic ankle strength test, both groups showed a significant increase in strength and power. In particular, eversion of the PLT showed significant improvement compared to that of the PBT at muscle powers of 30°/s and 120°/s. Patients with CAI have reported significantly lower inversion and eversion muscle strength than the general population. Hou et al. reported that when isokinetic muscle strength was assessed in 220 patients with CAI, both inversion and eversion were statistically significantly weaker [31]. The relationship between self-questionnaire scores for ankle stability and ultrasound scans of the peroneus longus and brevis muscles were previously tested in patients with CAI. The results showed that patients with high ankle instability had low strength in the peroneus longus and brevis muscles and a small cross-sectional area of the muscle [32]. Muscle strength and a decline in proprioceptive and eccentric motor control reportedly reduce the ability to control ankle inversion and this is a strong predictor of ankle re-sprain [33]. Prior research has highlighted the importance of both concentric and eccentric muscle contraction training in patients [25]. Training with the band in this study would have effectively improved muscle strength due to its ability to generate both concentric and eccentric actions [34].

One of the main results of this study concerned the Y-balance test, which evaluates the dynamic balance of the lower extremities. An interaction effect representing changes in both groups over time was confirmed in the posteromedial direction but not in the posterolateral direction. The peroneus longus muscle is considered necessary for ankle stability when the medial arch descends during posteromedial training. Subramanian et al. reported the possibility that ligaments or tendons around the ankles would not recover to their previous states if rehabilitation is not performed effectively in patients with CAI and that a decrease in the functional level of the ankle will negatively affect the functionality of the hip joint and other joints [35]. Therefore, the difference in Y-balance results can cause problems not only in the simple ankle but also in the entire lower extremity.

Smith et al. reported that there is a limit to strengthening the peroneus muscle in one direction and that the overall rehabilitation program should be considered by performing multi-directional ankle eversion considering the characteristics of the evertor [36]. As a countermeasure, Donnelly reported that the peroneus longus muscle is relatively activated when eversion is performed in the plantarflexion state and that the peroneus brevis muscle is most frequently used when eversion is performed in the neutral position [17].

This study has several clinical implications. A comparative analysis of independent training of the peroneus brevis, peroneus longus, and peroneus longus muscles showed superior pain reduction and functional improvement. Based on these findings, we suggest that eversion training should be performed in the plantarflexion position rather than in the existing neutral position in patients with CAI.

The study also has a few limitations. First, this study investigated the effect of exercise on men aged 30–50 years. Therefore, it is uncertain whether the same effect will be observed in athletes in their 20s and those involved in active sports. In this study, it was difficult to set up a control group because the participants were people who visited a rehabilitation center for treatment. Furthermore, there was a limit to controlling for personal training that deviated from the suggested training. In the future, investigating interventional studies by recruiting more participants from multiple institutions would be beneficial. In our study, the initial estimated sample size was 54, but 52 people were ultimately analyzed. These results may have potential for low explanatory power. Lastly, a critical limitation of this study is that the groups included only PLT and PBT, and there was no group that performed only stretching and cycling, nor was there a combined PLT and PBT group. Therefore, the interpretation of this study is that the PLT group only showed slightly greater improvements in eversion strength and balance ability than the PBT group, and it cannot not be stated that PLT is superior to PBT. Because PLT is rarely applied in the field, this study was conducted for simple comparison purposes. We would like to emphasize that the results and interpretations may vary in future studies conducted by various groups.

## 5. Conclusions

After conducting 12 weeks of training in patients with CAI, both PBT and PLT groups exhibited significant improvements in FAOS, isokinetic ankle strength, and Y-balance tests after the intervention compared to before the intervention. Regarding the effects observed in each group over time, it was observed that PLT was more effective than PBT in improving the strength and power of eversion and posterior medial balance. PLT exercise was shown to have a greater effect on both eversion strength and balance ability. Therefore, we recommend combining traditional PBT exercise with this study’s special PLT exercise.

## Figures and Tables

**Table 1:** General characteristics of participants.

Group	PBT (*n* = 26)	PLT (*n* = 26)	*t* or *x*^2^	*p*
Age (y)	40.8 ± 4.1	39.1 ± 4.8	0.851	0.548
Height (cm)	168.3 ± 7.5	169.4 ± 9.4	−0.323	0.623
Weight (kg)	76.8 ± 5.5	77.9 ± 6.8	−1.250	0.441
BMI	27.2 ± 3.8	27.1 ± 3.5	0.087	0.949
VAS	4.8 ± 2.1	4.6 ± 2.3	0.105	0.806
ROM (Dorsi-flexion), deg	19.9 ± 5.5	21.2 ± 4.5	−1.853	0.741
ROM (Plantar-flexion), deg	45.8 ± 7.7	45.1 ± 6.9	0.098	0.889
Injury side, left/right, *n*	14/12	15/12	0.158	0.802
Injury situation, *n*				
Sports or recreation	20	21	0.206	0.858
Walking on the street	5	3		
Stair	1	2		
Instability Grade				
Instability Grade I, *n*	7	8	0.548	0.760
Instability Grade II, *n*	13	11		
Instability Grade III, *n*	6	7		

Values are presented as mean ± standard deviation; PBT, Peroneus Brevis Training; PLT, Peroneus Longus Training; BMI, body mass index; VAS, Visual Analog Scale; ROM, Range of Motion.

**Table 2:** Changes in foot and ankle outcome score according to training.

Variables	PBT (*n* = 26)				PLT (*n* = 26)				Group × Time
	Pre	Post	Diff, %	*p*	Pre	Post	%Diff	*p*	*p*
Pain	68.1 ± 9.4	76.4 ± 11.6	12.2	0.003	66.4 ± 12.5	75.1 ± 13.6	13.1	0.015	0.427
Symptoms	70.8 ± 8.9	77.8 ± 10.1	9.9	0.010	69.9 ± 10.5	74.9 ± 10.1	7.2	0.007	0.235
Daily activities	67.3 ± 10.1	82.2 ± 9.9	22.1	0.038	69.5 ± 11.5	81.0 ± 11.1	16.5	<0.001	0.519
Sport and recreation	65.8 ± 7.1	79.8 ± 8.8	21.3	<0.001	67.5 ± 9.9	81.4 ± 10.7	20.6	<0.001	0.430
QoL	64.9 ± 7.9	76.2 ± 10.4	17.4	0.040	65.0 ± 8.7	75.9 ± 11.1	16.8	<0.001	0.458

*p* < 0.05; Values are presented as mean ± standard deviation; PBT, Peroneus Brevis Training; PLT, Peroneus Longus Training; QoL, quality of life; Diff, Difference.

**Table 3:** Changes in Isokinetic ankle joint function according to training.

Variables	PBT (*n* = 26)				PLT (*n* = 26)				Group × Time
	Pre	Post	Diff, %	*p*	Pre	Post	%Diff	*p*	*p*
30°/s inversion, Nm/kg, %	28.0 ± 6.6	35.2 ± 7.1	25.7	0.033	25.5 ± 5.4	33.2 ± 8.2	30.2	0.015	0.087
30°/s eversion, Nm/kg, %	32.7 ± 5.8	38.1 ± 5.9	16.5	0.015	29.8 ± 5.8	42.1 ± 6.4	41.4	<0.001	0.002
120°/s inversion, Watt/kg, %	14.2 ± 4.4	17.9 ± 6.7	26.3	0.020	15.6 ± 3.4	18.2 ± 4.9	16.7	0.031	0.520
120°/s eversion, Watt/kg, %	16.4 ± 5.5	18.8 ± 8.7	14.8	0.040	16.5 ± 5.2	22.2 ± 6.9	34.2	<0.001	0.005

*p* < 0.05; Values are presented as mean ± standard deviation; PBT, Peroneus Brevis Training; PLT, Peroneus Longus Training; %Diff., Diff, Difference.

**Table 4:** Changes in Y-balance test according to training.

Variables	PBT (*n* = 26)				PLT (*n* = 26)				Group × Time
	Pre	Post	Diff, %	*p*	Pre	Post	%Diff	*p*	*p*
AT (cm)	53.7 ± 11.4	59.9 ± 10.0	11.5	0.015	55.0 ± 10.8	64.3 ± 14.6	16.9	0.003	0.022
PM (cm)	78.6 ± 18.4	83.6 ± 18.9	6.4	0.080	81.3 ± 16.7	92.0 ± 20.2	13.1	0.033	<0.001
PL (cm)	74.8 ± 18.0	85.0 ± 20.7	13.5	0.048	75.0 ± 20.1	85.7 ± 21.4	14.2	0.030	0.800
YBT score	78.2 ± 15.1	87.4 ± 19.0	11.7	0.033	77.8 ± 16.2	88.0 ± 17.8	13.1	0.021	0.780

*p* < 0.05; Values are presented as mean ± standard deviation; PBT, Peroneus Brevis Training; PLT, Peroneus Longus Training; AT, Anterior; PM, Posteromedial; PL: Posterolateral; YBT score, Diff., Difference.

## Data Availability

The data presented in this study are available on request from the corresponding authors.

## Supplementary Materials

The following supporting information can be downloaded at: https://www.mdpi.com/article/10.3390/healthcare12050547/s1, Table S1: CONSORT 2010 checklist of information to include when reporting a randomised trial.

## Abbreviations

ANOVA	Analysis of variance
ATFL	Anterior talofibular ligament
BMI	Body mass index
CAI	Chronic ankle instability
EMG	Electromyography
FAOS	Foot and ankle outcome score
PBT	Peroneus Brevis Training
PLT	Peroneus Longus Training
PTFL	Posterior talofibular ligament
RPE	Rating Perceived Exertion

## References

[1] Dubin A. (2014). Gait: The role of the ankle and foot in walking. Med. Clin..

[2] Herzog M.M., Kerr Z.Y., Marshall S.W., Wikstrom E.A. (2019). Epidemiology of ankle sprains and chronic ankle instability. J. Athl. Train..

[3] Medina McKeon J.M., Hoch M.C. (2019). The ankle-joint complex: A kinesiologic approach to lateral ankle sprains. J. Athl. Train..

[4] Zhang J., Yang K., Wang C., Gu W., Li X., Fu S., Song G., Wang J., Wu C., Zhu H. (2023). Risk factors for chronic ankle instability after first episode of lateral ankle sprain: A retrospective analysis of 362 cases. J. Sport Health Sci..

[5] Bhimani R., Sato G., Saengsin J., Lubberts B., Waryasz G., DiGiovanni C.W., Guss D. (2022). Fluoroscopic Evaluation of the Role of Syndesmotic Injury in Lateral Ankle Instability in a Cadaver Model. J. Foot Ankle Int..

[6] Hegazy M.A., Khairy H.M., Hegazy A.A., Sebaei M.A.E.F., Sadek S.I. (2023). Talus bone: Normal anatomy, anatomical variations and clinical correlations. J. Anat. Sci. Int..

[7] Park B.S., Chung C.Y., Park M.S., Sung K.H., Choi Y., Park C., Koo S., Lee K.M. (2019). Inverse relationship between radiographic lateral ankle instability and osteochondral lesions of the talus in patients with ankle inversion injuries. J. Foot Ankle Int..

[8] Lysdal F.G., Wang Y., Delahunt E., Gehring D., Kosik K.B., Krosshaug T., Li Y., Mok K.-M., Pasanen K., Remus A. (2022). What have we learnt from quantitative case reports of acute lateral ankle sprains injuries and episodes of ‘giving-way’of the ankle joint, and what shall we further investigate?. J. Sports Biomech..

[9] Lambert L.-A., Falconer L., Mason L. (2020). Ankle stability in ankle fracture. J. Clin. Orthop. Trauma.

[10] Ma T., Li Q., Song Y., Hua Y. (2021). Chronic ankle instability is associated with proprioception deficits: A systematic review and meta-analysis. J. Sport Health Sci..

[11] Seok H., Lee S.H., Yun S.J. (2020). Diagnostic performance of ankle ultrasound for diagnosing anterior talofibular and calcaneofibular ligament injuries: A meta-analysis. J. Acta Radiol..

[12] Boey H., van Rossom S., Verfaillie S., Vander Sloten J., Jonkers I. (2022). Maximal lateral ligament strain and loading during functional activities: Model-based insights for ankle sprain prevention and rehabilitation. J. Clin. Biomech..

[13] Pereira B.S., Andrade R., Espregueira-Mendes J., Marano R.P.C., Oliva X.M., Karlsson J. (2021). Current concepts on subtalar instability. Orthop. J. Sports Med..

[14] Rodrigues K.A., Soares R.J., Tomazini J.E. (2019). The influence of fatigue in evertor muscles during lateral ankle sprain. J. Foot.

[15] Mendez-Rebolledo G., Guzmán-Venegas R., Valencia O., Watanabe K. (2021). Contribution of the peroneus longus neuromuscular compartments to eversion and plantarflexion of the ankle. PLoS ONE.

[16] Donnelly L., Donovan L., Hart J.M., Hertel J. (2017). Eversion strength and surface electromyography measures with and without chronic ankle instability measured in 2 positions. Foot Ankle Int..

[17] Koldenhoven R.M., Feger M.A., Fraser J.J., Saliba S., Hertel J. (2016). Surface electromyography and plantar pressure during walking in young adults with chronic ankle instability. Knee Surg. Sports Traumatol. Arthrosc..

[18] Chen E.T., McInnis K.C., Borg-Stein J. (2019). Ankle sprains: Evaluation, rehabilitation, and prevention. Curr. Sports Med. Rep..

[19] Ahn S.-h., Hwang U.-j., Gwak G.-t., Yoo H.-i., Kwon O.-y. (2020). Comparison of the strength and electromyography of the evertor muscles with and without toe flexion in patients with chronic ankle instability. Foot Ankle Int..

[20] Li F., Kong Z., Zhu X., Chow B.C., Zhang D., Liang W., Shang B., Liu Y., Zhang H. (2022). High-intensity interval training elicits more enjoyment and positive affective valence than moderate-intensity training over a 12-week intervention in overweight young women. J. Exerc. Sci. Fit..

[21] Wen H.-J., Liu S.-H., Tsai C.-L. (2022). Effects of 12 weeks of aerobic exercise combined with resistance training on neurocognitive performance in obese women. J. Exerc. Sci. Fit..

[22] Goulart Neto A.M., Maffulli N., Migliorini F., de Menezes F.S., Okubo R. (2022). Validation of Foot and Ankle Ability Measure (FAAM) and the Foot and Ankle Outcome Score (FAOS) in individuals with chronic ankle instability: A cross-sectional observational study. J. Orthop. Surg. Res..

[23] Sierra-Guzmán R., Jiménez F., Abián-Vicén J. (2018). Predictors of chronic ankle instability: Analysis of peroneal reaction time, dynamic balance and isokinetic strength. Clin. Biomech..

[24] Powden C.J., Dodds T.K., Gabriel E.H. (2019). The reliability of the star excursion balance test and lower quarter Y-balance test in healthy adults: A systematic review. Int. J. Sports Phys. Ther..

[25] Picot B., Terrier R., Forestier N., Fourchet F., McKeon P.O. (2021). The star excursion balance test: An update review and practical guidelines. J. Int. J. Athl. Ther. Train..

[26] Sarcon A.K., Heyrani N., Giza E., Kreulen C. (2019). Lateral ankle sprain and chronic ankle instability. Foot Ankle Orthop..

[27] Park Y.H., Park S.H., Kim S.H., Choi G.W., Kim H.J. (2019). Relationship between isokinetic muscle strength and functional tests in chronic ankle instability. J. Foot Ankle Surg..

[28] Cain M.S., Ban R.J., Chen Y.-P., Geil M.D., Goerger B.M., Linens S.W. (2020). Four-week ankle-rehabilitation programs in adolescent athletes with chronic ankle instability. J. Athl. Train..

[29] Park Y.-J., Cho Y.-H., Seo T.-B. (2023). Effect of two different exercises on balance, pain and ankle motor function in male college students with chronic ankle instability. J. Men’s Health.

[30] Burkhard M.D., Wirth S.H., Andronic O., Viehöfer A.F., Imhoff F.B., Fröhlich S. (2021). Clinical and functional outcomes of peroneus longus to brevis tendon transfer. Foot Ankle Int..

[31] Hou Z.-C., Miao X., Ao Y.-F., Hu Y.-L., Jiao C., Guo Q.-W., Xie X., Zhao F., Pi Y.-B., Li N. (2020). Characteristics and predictors of muscle strength deficit in mechanical ankle instability. BMC Musculoskelet. Disord..

[32] ÖZGÜL B., STARBUCK C., POLAT M.G., ABDEEN R., NESTER C. (2020). Relationship between self-reported functional stability and peroneal muscle structure in individuals with chronic ankle instability. J. Exerc. Ther. Rehabil..

[33] Delahunt E., Remus A. (2019). Risk factors for lateral ankle sprains and chronic ankle instability. J. Athl. Train..

[34] Wang B., Zhang X., Zhu F., Zhu W., Wang X., Jia F., Chen W., Zhang M. (2022). A randomized controlled trial comparing rehabilitation with isokinetic exercises and Thera-Band strength training in patients with functional ankle instability. PLoS ONE.

[35] Subramanian S.S., Teng S.Y., Senthil P., Gaowgeh R.A.M., Alfawaz S.S., Neamatallah Z. (2021). Correlation between Level of Functional Ability and Sports Performance among Football Players with Chronic Ankle Instability (CAI). J. Pharm. Res. Int..

[36] Smith B.I., Docherty C.L., Simon J., Klossner J., Schrader J. (2012). Ankle strength and force sense after a progressive, 6-week strength-training program in people with functional ankle instability. J. Athl. Train..

